# Supplementary material for: Effects of a Home-Based Lifestyle Intervention Program on Cardiometabolic Health in Breast Cancer Survivors during the COVID-19 Lockdown
Source: J Clin Med. 2021 Jun 17;10(12):2678. doi: 10.3390/jcm10122678 (PMC8235209; doi:10.3390/jcm10122678)
Supplement: Supplementary file 1 [file jcm-10-02678-s001.zip › Supplementary Figure S1.pdf]

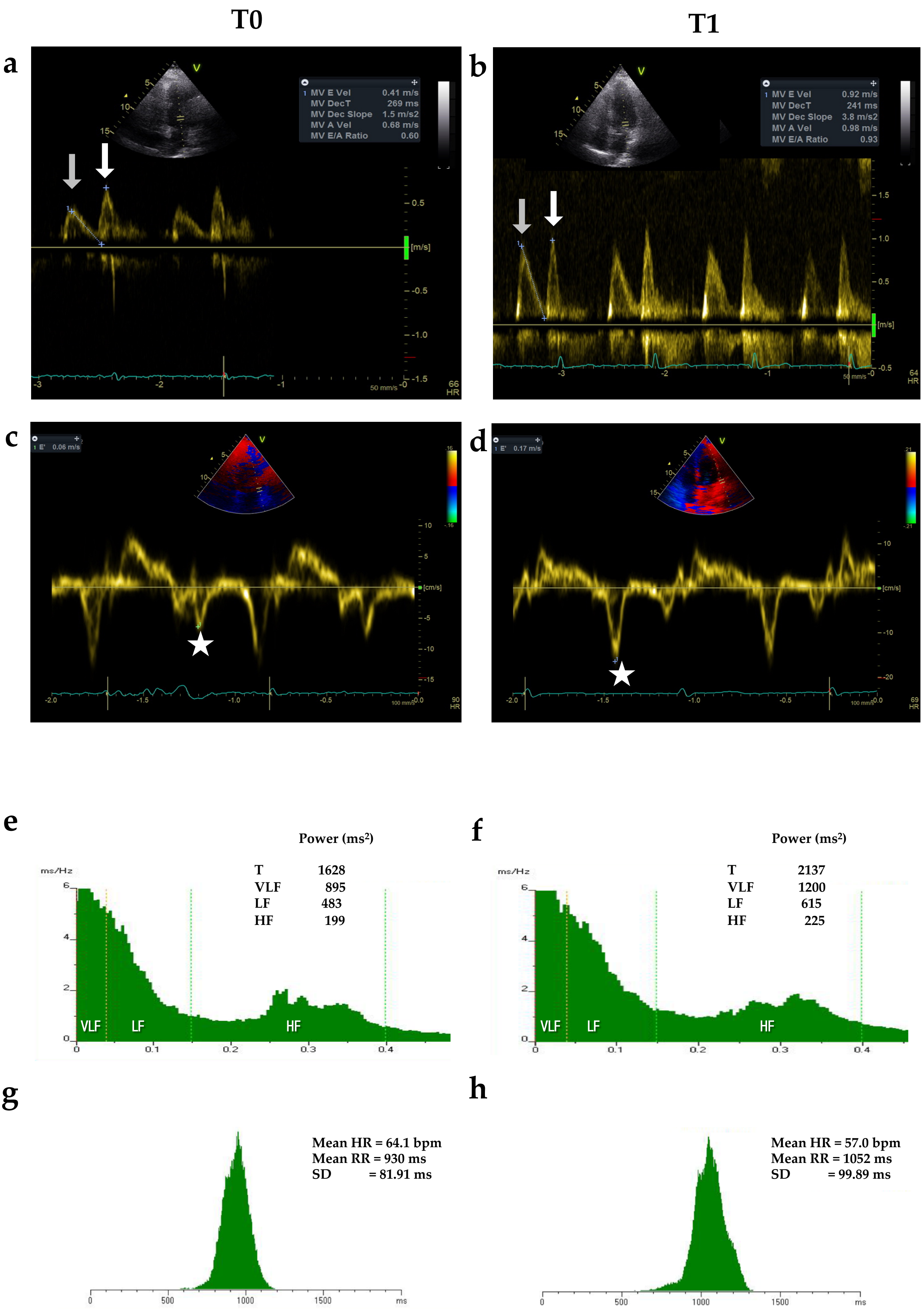

T0
T1
a
MV E Vel 0.41 m/s
MV DecT 269 ms
MV Dec Slope 1.5 m/s2
MV A Vel 0.68 m/s
MV E/A Ratio 0.60
b
MV E Vel 0.92 m/s
MV DecT 241 ms
MV Dec Slope 3.8 m/s2
MV A Vel 0.98 m/s
MV E/A Ratio 0.93
c
E' 0.06 m/s
d
E' 0.17 m/s
e
Power (ms²)
T 1628
VLF 895
LF 483
HF 199
VLF
LF
HF
f
Power (ms²)
T 2137
VLF 1200
LF 615
HF 225
VLF
LF
HF
g
Mean HR = 64.1 bpm
Mean RR = 930 ms
SD = 81.91 ms
h
Mean HR = 57.0 bpm
Mean RR = 1052 ms
SD = 99.89 ms

Figure S1. Changes in representative cardiovascular parameters. Left panel shows baseline (T0) evaluation. Right panel shows the same parameters after 3-month (T1). Black-background figures are echocardiographic parameters, white background 24-h derived parameters. A mitral early inflow (E wave, grey arrow) is reduced and broader compared to atrial inflow (A wave, white arrow) with an E/A peak velocity ratio of 0.7, sign of grade 1 diastolic dysfunction. C mitral annulus peak velocity by tissue doppler (E′ wave, star) is 0.06 m/s (reduced). B and D after three months there is an improvement in E/A ratio to 0.93 and an increase of E' to 0.17 m/s (normalization of diastolic function). E HRV frequency bands divided in VLF, LF and HF (vertical dotted line). Total power sum of each frequency band. At T1 (F) there is an increase in total power, mainly due to increase in VLF and LF. G histogram of total beats is shown with mean HR of 64 bpm with a SD of total intervals of 81.9 ms. At T1 (H) the histogram is wider due to an increase in SD (99.8 ms) and shifted rightward (decrease of mean HR to 57 bpm). HRV heart rate variability, VLF very low frequency, LF low frequency, HF high frequency, SD standard deviation.
