# Supplementary material for: Effects of a Home-Based Lifestyle Intervention Program on Cardiometabolic Health in Breast Cancer Survivors during the COVID-19 Lockdown
Source: J Clin Med. 2021 Jun 17;10(12):2678. doi: 10.3390/jcm10122678 (PMC8235209; doi:10.3390/jcm10122678)
Supplement: Supplementary file 1 [file jcm-10-02678-s001.zip › Supplementary Table S1.pdf]

Table S1. Multivariate general linear model's F(*p*) values, which show the multivariate and univariate effects of the interaction between time (pre- and post-intervention) and study arm (intervention and control) on several representative parameters.

	**Multivariate**	**Univariate post-hoc**				
		BMI	Glycemia	Triglycerides	LDL	$\dot{V}O_{2max}$
Interaction effect	0.664 (0.655)	2.721 (0.112)	1.223 (0.279)	0.414 (0.526)	0.640 (0.431)	0.343 (0.563)

BMI, body mass index; LDL, low-density lipoprotein; $\dot{V}O_{2max}$, maximal oxygen uptake.
